# Identifying optimal vaccination scenarios to reduce varicella zoster virus transmission and reactivation

**DOI:** 10.1186/s12916-022-02534-7

**Published:** 2022-10-08

**Authors:** Kevin M Bakker, Marisa C Eisenberg, Robert J Woods, Micaela E Martinez

**Affiliations:** 1 Department of Epidemiology, University of Michigan, 48109 Ann Arbor, MI USA; 2 Department of Mathematics, University of Michigan, 48109 Ann Arbor, MI USA; 3 Division of Infectious Diseases, Department of Internal Medicine, University of Michigan, 48109 Ann Arbor, MI USA; 4 Population Biology, Ecology and Evolution, Emory University, 30322 Atlanta, GA USA; 5 University of Surrey, Faculty of Health and Medical Sciences, Guildford, UK

**Keywords:** Varicella zoster virus, Chickenpox, Shingles, Vaccination, Mathematical modeling

## Abstract

**Background:**

Varicella zoster virus (VZV) is one of the eight known human herpesviruses. Initial VZV infection results in chickenpox, while viral reactivation following a period of latency manifests as shingles. Separate vaccines exist to protect against both initial infection and subsequent reactivation. Controversy regarding chickenpox vaccination is contentious with most countries not including the vaccine in their childhood immunization schedule due to the hypothesized negative impact on immune-boosting, where VZV reactivation is suppressed through exogenous boosting of VZV antibodies from exposure to natural chickenpox infections.

**Methods:**

Population-level chickenpox and shingles notifications from Thailand, a country that does not vaccinate against either disease, were previously fitted with mathematical models to estimate rates of VZV transmission and reactivation. Here, multiple chickenpox and shingles vaccination scenarios were simulated and compared to a model lacking any vaccination to analyze the long-term impacts of VZV vaccination.

**Results:**

As expected, simulations suggested that an introduction of the chickenpox vaccine, at any coverage level, would reduce chickenpox incidence. However, chickenpox vaccine coverage levels above 35% would increase shingles incidence under realistic estimates of shingles coverage with the current length of protective immunity from the vaccine. A trade-off between chickenpox and shingles vaccination coverage was discovered, where mid-level chickenpox coverage levels were identified as the optimal target to minimize total zoster burden. Only in scenarios where shingles vaccine provided lifelong immunity or coverage exceeded current levels could large reductions in both chickenpox and shingles be achieved.

**Conclusions:**

The complicated nature of VZV makes it impossible to select a single vaccination scenario as universal policy. Strategies focused on reducing both chickenpox and shingles incidence, but prioritizing the latter should maximize efforts towards shingles vaccination, while slowly incorporating chickenpox vaccination. Alternatively, countries may wish to minimize VZV complications of both chickenpox and shingles, which would lead to maximizing vaccine coverage levels across both diseases. Balancing the consequences of vaccination to overall health impacts, including understanding the impact of an altered mean age of infection for both chickenpox and shingles, would need to be considered prior to any vaccine introduction.

**Supplementary Information:**

The online version contains supplementary material available at 10.1186/s12916-022-02534-7.

## Background

Varicella zoster, commonly referred to as chickenpox, is a respiratory infectious disease that causes a characteristic red rash and pox on the skin surface [1]. It is caused by the varicella zoster virus (VZV) which also causes shingles, often referred to as herpes zoster. Chickenpox symptoms typically arise 1–3 weeks after exposure to an infected individual, and a newly infected individual is infectious for around a week starting 1–2 days prior to the onset of symptoms. Symptoms last approximately 2 weeks, when the virus then retreats to the nerve ganglia in the spine [2, 3]. By the age of 15, nearly all children have antibodies to VZV, whether from a natural infection or vaccine [4, 5]. In 10–30% of adults, the latent varicella virus will reactivate and manifest as shingles, typically in adults aged over 60 [6, 7]. The VZV vaccine prevents infection with VZV in children while a booster dose, referred to as a shingles vaccine, later in life suppresses VZV reactivation in adults [4].

The chickenpox vaccine was first approved for use in the USA in 1995. Despite its successful use for a quarter century, global VZV vaccination policy remains a topic of much debate. It is a live-attenuated vaccine administered in two doses during childhood. The shingles vaccine is either a single (Zostovax) or double (Shingrix) dose recombinant vaccine given later-in-life to suppresses reactivation [4, 8, 9]. Chickenpox vaccination is only used in a limited number of countries, and there are multiple reasons why most countries have not yet implemented vaccination. First, complications from chickenpox are rare, with less than 1% of infected individuals experiencing severe illness [10]. Second, childhood immunization against VZV has been hypothesized to reduce natural VZV exposure in adults. This reduction of VZV exposure could then reduce VZV immune-boosting, which would cause additional reactivation [11–16]. Third, low and intermediate levels of chickenpox vaccine coverage would shift the age distribution of chickenpox infection onto older age groups who would carry a higher burden of disease [17]. Severe complications become more common in individuals who contract chickenpox at an older age [18, 19]. Fourth, as with other vaccine-preventable diseases, it is likely that natural infection provides longer immunity than a vaccine dose [20, 21]. Despite the complexities surrounding VZV vaccination, some countries have chosen to vaccinate. Although the percent of infections resulting in serious illness is low, when endemic VZV infects the majority, vaccination prevents a substantial number of serious cases (e.g., hospitalizations). For instance, vaccination prevents an estimated 4 million cases each year in the United States [22], and 1% of those averted cases (approximately 40,000) would have been serious illness.

The primary reason the VZV vaccine has not been implemented worldwide is the presumed reduction of immune-boosting in adults. Theoretical models have predicted an increase in shingles incidence with the inclusion of the chickenpox vaccine on a countries childhood immunizations schedule. [23, 24]. Importantly, these models are theoretical and have not been fit to, or challenged with, data. Out of necessity they have been developed in the face of many unknowns regarding viral latency. Immunity boosting has been used as a general term for the reinforcement of VZV-specific immunity, which is most likely dominated by cellular immunity. It is this T cell-mediated immunity that protects from VZV reactivation [15]. However, empirical evidence from surveillance programs in locations that vaccinate against chickenpox have been inconclusive about the impact of chickenpox vaccination on shingles. There have been both observed increases [25–27] and no change [28, 29] in shingles incidence.

It is difficult to discern whether increases in shingles are due to the vaccine or improved reporting. Prior to the introduction of the chickenpox vaccine, cases of shingles had been increasing in Canada, [30, 31], the UK [30], and the USA [32]. In Spain, where chickenpox vaccination occurs in a limited geographic areas (e.g., Madrid but nowhere else on the mainland [33]), shingles incidence has been steadily increasing due to demographic changes [34]. With the global availability of the VZV vaccine, and other herpesvirus vaccines in development [35–37], it is vital to understand the long-term impacts of any vaccine introduction. Data from Thailand were selected for this simulation study due to the availability of population level chickenpox and shingles data. Multiple immunization scenarios were examined in Thailand, which does not vaccinate, to interpret the long-term dynamics of chickenpox and shingles.

## Results

### Chickenpox dynamics following vaccination

**Fig. 1:**
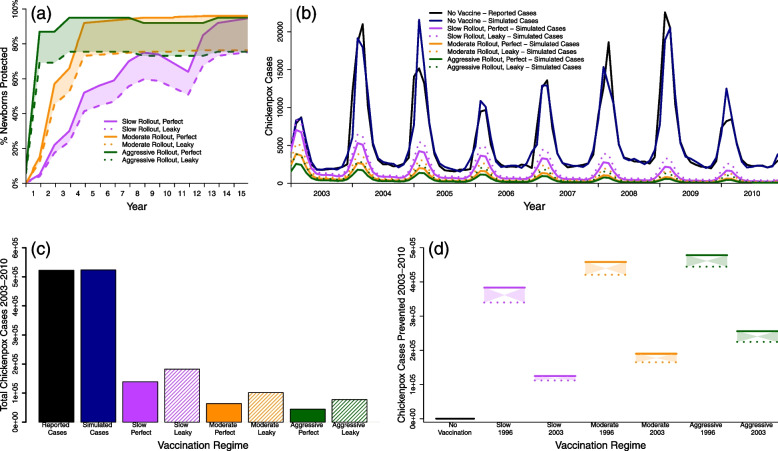
Best fit model simulations under various immunization approaches. For all panels; reported chickenpox cases (black), simulated chickenpox cases without vaccination (blue), simulated chickenpox cases utilizing the data from the (slow) 1984 measles vaccine roll-out in Thailand (magenta), simulated chickenpox cases utilizing the data from the (moderate) 1992 hepatitis B, 3rd dose vaccine roll-out in Thailand (orange), and simulated chickenpox cases utilizing the (aggressive) 2006 Japanese Encephalitis roll-out in Thailand (green). **a** Uptake levels for the 3 roll-outs with perfect (solid line) and leaky (dashed line) uptake. Colored regions between dashed and solid lines represent realistic ranges of coverage. **b** Time series of simulated cases under various immunization roll-outs, during our study period (2003–2010) if vaccination had started in 1996. **c** Total reported, simulated, and immunization estimates for chickenpox cases over our 8 year study period (2003–2010). **d** Chickenpox cases prevented under various conditions, including if vaccination had started in 1996 or 2003. *X*-axis labels represent vaccine coverage, vaccine start date, with dashed lines representing leaky vaccines and solid representing perfect vaccines

To evaluate the impact of chickenpox vaccination, we estimated the number of cases that would have been averted had the chickenpox vaccine had been introduced in Thailand’s routine immunization program in 1996 under various roll-out scenarios (Fig. 1). The simulated model without vaccination closely resembled the raw data (Fig. 1b, c). Model simulations that included immunization revealed a large drop in the number of chickenpox cases (Fig. 1b–d). Routine infant immunization was implemented by vaccinating a portion of newborns each year, a value that varied depending on coverage and uptake (Fig. 1a).

**Fig. 2:**
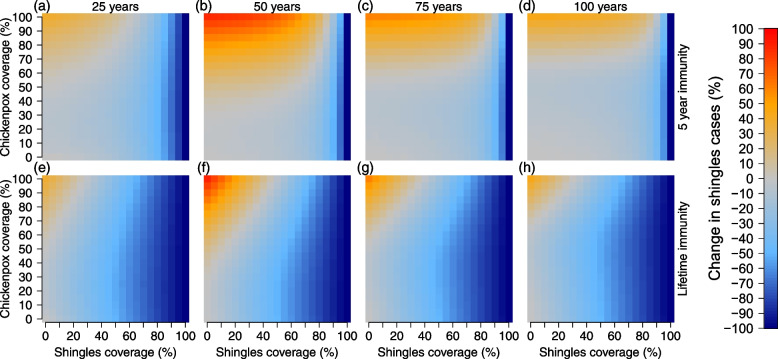
Percent change in shingles cases compared to no vaccination, using a 20 year protection from chickenpox vaccination. Top row (**a**–**d**) simulations provided 5 years of immunity from shingles vaccination and bottom row (**e**–**h**) simulations provided lifetime immunity from shingles vaccination. Each column represents the number of years after vaccine introduction: **a** and **e** 25 years, **b** and **f** 50 years, **c** and **g** 75 years, and **d** and **h** 100 years. Shingles and chickenpox vaccine coverage (%) are shown on the *x*- and *y*-axes. Color scale on the right indicates the largest decrease in shingles cases can be seen in dark blue, while the largest increase in shingles cases can be seen in red

If vaccination in Thailand had begun in 1996, a year after the US licensed the chickenpox vaccine, the model estimated that between 340,000 and 480,000 chickenpox cases would have been prevented, representing a 65–91.6% reduction in cases, during the period 2003–2010 (period for which we fit the original model) [38]. With a slow leaky roll-out, where newborn protection did not reach 50% until the 7th year of the program, and 75% until the 15th year [39] (Fig. 1a), 340,000 cases of chickenpox would have been prevented. A more aggressive vaccine roll-out and higher efficacy (i.e., that of the Japanese Encephalitis with perfect uptake), would have prevented nearly 480,000 cases during the same time period, signifying a $91.6\%$ reduction in cases. These results revealed a non-linear relationship between vaccination and reported cases, as a proportional increase in vaccine coverage did not further prevent an equal proportion of chickenpox cases. This relationship is further explored in Fig. S1. Simulations were intentionally conservative by only immunizing newborns, so any VZV immunization catch-up efforts would further reduce the number of chickenpox cases.

By the end of the 100-year simulation period, chickenpox cases were near zero under any of the vaccination coverage and uptake combinations (Tables S2 and S3). All vaccination scenarios cut the number of chickenpox cases by at least half in the first 25 years, at least 94% in the second 25 years, and 98% in the years 50–100 of the simulations which equated to less than a dozen cases monthly across the entire country.

**Fig. 3:**
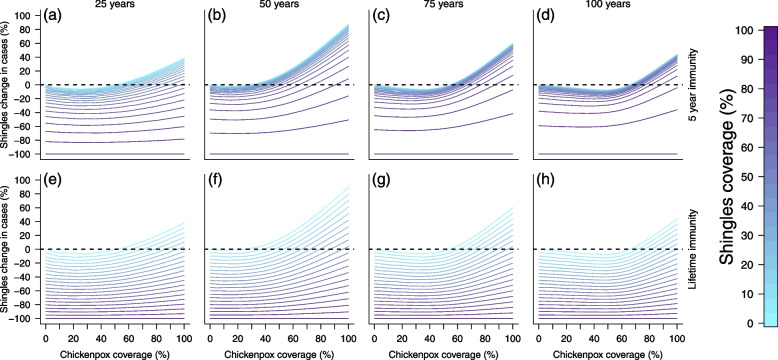
Cross section of Fig. 2. Top row represents simulations with 5 years of shingles vaccine immunity and the bottom row represents simulations with lifetime shingles vaccine immunity. Each column visualizes vaccination impact after 25 (left), 50 (middle left), 75 (middle right), and 100 (right) years. *X*-axes represent the chickenpox vaccination coverage, while the *y*-axes represents the change in shingles cases from the null vaccination simulation. Colors represent shingles vaccination coverage with a gradient from no vaccination (light blue) to fully vaccinated (dark purple). Dotted line at 0 identifies where there would be an increase (above the line) or decrease (below the line) in shingles cases compared to the simulation lacking vaccination

**Fig. 4:**
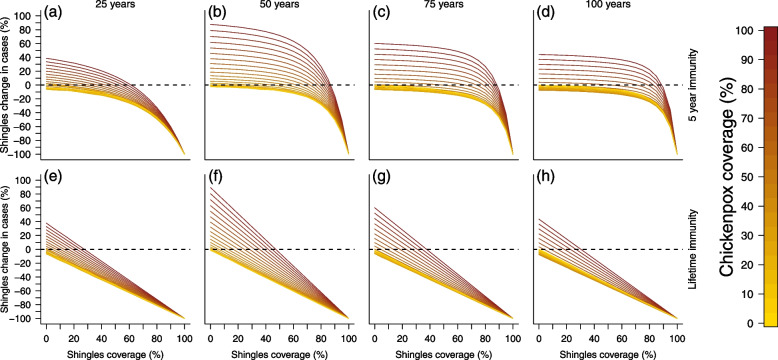
Cross section of Fig. 2. Top row represents simulations with 5 years of shingles vaccine immunity and the bottom row represents simulations with lifetime shingles vaccine immunity. Each column visualizes vaccination impact after 25 (left), 50 (middle left), 75 (middle right), and 100 (right) years. *X*-axes represent the shingles vaccination coverage, while the *y*-axes represents the change in shingles cases from the null vaccination simulation. Colors represent chickenpox vaccination coverage with a gradient from no vaccination (yellow) to fully vaccinated (dark red). Dotted line at 0 identifies where there would be an increase (above the line) or decrease (below the line) in shingles cases compared to the simulation lacking vaccination

### Shingles dynamics following vaccination

Shingles dynamics were more complex due to chickenpox vaccination increasing the number of individuals susceptible to VZV reactivation after the initial 20 year protective period from chickenpox vaccination. Vaccine simulations were evaluated against a null simulation which lacked both chickenpox and shingles vaccination. To examine the impact of vaccination at different points of the 100-year simulation, we separated the results into four 25-year time periods (Fig. S2 and Tables S2 and S3). Over the 100 year period, scenarios that included lifetime shingles protection from vaccination revealed the greatest reduction in shingles cases. For the simulations that examined shingles dynamics with only 5 years of immunity from vaccination, high shingles coverage levels reduced shingles cases the greatest over the 100 year period. Simulations revealed that greater reductions in shingles cases occurred from leaky or low chickenpox coverage (any roll-out). Importantly, the model identified a trade-off in chickenpox and shingles coverage on shingles dynamics. This relationship revealed unexpected scenarios where mid-range chickenpox coverage was the best long-term strategy for reducing shingles cases under realistic shingles vaccination levels (Figs. 2, 3, and 4). Mid-range chickenpox coverage was also identified as the most effort-effective strategy for reducing chickenpox cases (Fig. S1).

Unsurprisingly, most scenarios that included shingles vaccination decreased shingles cases in the first period (years 1–25) of our simulation. Only when chickenpox coverage was high and shingles coverage was low did shingles cases increase in this period. This is because the length of immunity from chickenpox vaccination lasted 20 years, so the increase in cases was driven by those losing immunity in the last few years of this period. In the second and third periods (years 25-75) all scenarios saw in increase in shingles cases compared to the first period. In the final period, all scenarios saw a drop in shingles cases compared to the second and third periods (Figs. 2, S2, and Table S2).

## Discussion

In this study, mathematical models fit to chickenpox and shingles data from Thailand were used to simulate various vaccination scenarios to (i) reveal that any introduction of a chickenpox vaccine would drastically reduce chickenpox incidence; (ii) identify a non-linear relationship in chickenpox coverage and reduction of chickenpox cases; (iii) demonstrate that any introduction of a shingles vaccine with realistic coverage levels ($\le$ 50%), in combination with realistic chickenpox coverage levels ($\ge$ 35%) would increase shingles incidence, unless the immunity provided from shingles vaccination was lifelong; and (iv) uncover a trade-off in chickenpox and shingles vaccine coverage on shingles incidence. The lack of population-level shingles data had previously limited VZV vaccination policy research to theoretical models or models based on small-sample sizes. Here, the dynamical implications of different vaccination scenarios were examined using models fit to population-level chickenpox and shingles data.

Simulations of chickenpox vaccination in Thailand demonstrated the potential for up to a $91.6\%$ drop in chickenpox cases during the initial 8-year study period (Fig. 1). These results were derived from realistic scenarios reflecting previous immunization efforts in Thailand, and are in-line with the US experience with VZV vaccination, which had a 67–84% reduction in chickenpox cases after only 5 years of immunization [40]. As expected, increased chickenpox vaccine coverage reduced the total number of chickenpox cases; additionally, even a gradual vaccine roll-out would drastically reduce chickenpox morbidity if higher coverage levels were not feasible, which is evidence of herd immunity [41, 42]. Simulations also revealed that low-to-medium chickenpox vaccination efforts would have larger than expected impacts on reducing chickenpox incidence, while higher vaccination efforts would have reduced effects on chickenpox incidence (Fig. S1). There were minimal differences between the three chickenpox roll-out scenarios over the course of the 100-year simulation; in all scenarios chickenpox cases fell below 2% of the null vaccination scenario after 50 years. Our model assumed heterogeneous mixing, and halfway through the 100-year simulation there were less than 50 cases of chickenpox annually across the country under all vaccination scenarios. It is likely that chickenpox would stochastically die off well before this point due to low numbers and herd immunity, with occasional re-introductions to pockets of under-vaccinated populations [43].

Under realistic scenarios of shingles vaccination (up to 50% coverage with 5 years of protective immunity), simulations that included slow, moderate, or aggressive chickenpox vaccine roll-out increased shingles incidence (Fig. S2), though some scenarios of lower chickenpox coverage resulted in slight shingles case reductions (Fig. 2). This was because immunity from chickenpox vaccination only provided 20 years of protection, which increased the number of individuals available for shingles reactivation. The only simulations where vaccination had a noticeable long-term impact on shingles reduction compared to no vaccination occurred when immunity from shingles coverage was vaccination was lifelong or when immunity was 5 years and shingles coverage was extremely high. Even with lifetime immunity from shingles vaccination, there were no scenarios where realistic shingles coverage equated to an equal rate of case reduction when chickenpox vaccination was also included (Tables S2 and S3). Only when chickenpox vaccination was removed and shingles immunity was lifelong did vaccinating half (UK estimate) or a third (US estimate) of the population reduce the total shingles cases by 50% or 33% (Fig. 4). While scenarios with lower chickenpox coverage/coverage and high shingles coverage with lifetime immunity performed best, we observed an interesting trade-off between chickenpox uptake and shingles coverage, which existed in both the 5 year and lifetime shingles immunity simulations (Figs. 2 and S2).

Under realistic vaccination scenarios observed in countries that currently vaccinate against both chickenpox and shingles, where the shingles vaccine provides 5 years of immunity and high chickenpox coverage ($\ge$ 50%) exists, anything less than 80% shingles coverage led to an increase in shingles incidence compared to the null model at some point in the simulation (Figs. 2 and 3). This high level of shingles coverage would be nearly impossible to achieve at the population level, as routine adult immunizations are uncommon and the highest cited level of national shingles coverage is 50% [44–46]. Under both the 5 year and lifetime immunity from shingles vaccination simulations, mid-level chickenpox coverage resulted in a greater reduction in shingles incidence than low or high chickenpox coverage. This ‘C’ shape can be seen in Figs. 2 and 3 (seen as a “U”). If chickenpox vaccine immunity was extended to 40 years, this pattern remained, though the increase in shingles cases at higher chickenpox vaccination levels was reduced (Fig. S3). These counter-intuitive results reveal that to achieve the greatest reduction in both chickenpox and shingles policy makers should strive for mid-level chickenpox coverage and focus their efforts on maximizing shingles coverage.

An important limitation of this work, which examined chickenpox and shingles dynamics under various vaccination scenarios in Thailand, was that it did not include infection complications, including VZV caused death in the model. Any low- to mid-level chickenpox coverage would lead to an increase in the mean age of chickenpox infection, which could lead to more serious chickenpox complications in unvaccinated individuals if herd immunity were not rapidly achieved and sustained. However, while chickenpox vaccination would increase shingles incidence, previous work has demonstrated that chickenpox vaccination would reduce the mean age of shingles reactivation [47], potentially curtailing serious side-effects of shingles, as younger individuals tend to have milder symptoms [48–51].

Furthermore all simulations omitted the potential impact of chickenpox vaccination reducing population-level VZV exogenous boosting in adults. Any reduction of this boosting (via chickenpox vaccination) would likely increase the number of shingles cases, particularly at higher chickenpox vaccination levels seen in Figs. 2, 3, 4, and S3. We previously attempted to fit exogenous boosting to these data from Thailand [38], and models that included boosting did not perform better. These are vital next steps in VZV vaccination research. There have been a host of theoretical models attempting to understand the impact of such boosting, however population-level studies remain sparse, primarily because shingles is not a notifiable disease in most countries. Important questions regarding exogenous boosting include; What is the relationship between chickenpox vaccination and exogenous VZV boosting? At what levels of population-level chickenpox vaccination coverage do we start to see an impact (decrease) in population-level VZV immunity? Is this relationship linear or exponential? Does chickenpox vaccination impact the length of immunity from VZV reactivation differently from individuals with a natural infection?

## Conclusions

The complicated nature of VZV makes it impossible to select a single vaccination scenario as universal policy. Some countries may wish to minimize total VZV cases, while others may prefer to focus on chickenpox or shingles individually. Strategies focused on reducing both chickenpox and shingles incidence, but prioritizing the latter, should concentrate on raising awareness for shingles vaccination [45] and maximize efforts towards shingles vaccination, while slowly incorporating chickenpox vaccination. The observed non-linear relationship between chickenpox coverage and the number of cases prevented could be exploited to minimize both chickenpox and shingles incidence. Low and high chickenpox vaccine coverage performed similarly in preventing chickenpox cases during the first few years of the simulation (Fig. 1), and were nearly identical in the long term (Table S2 and S3) while lower chickenpox coverage also prevented excess shingles cases (Fig. S2). Alternatively, countries may wish to minimize VZV complications of both chickenpox and shingles, which would lead to maximizing vaccination across both chickenpox and shingles. Balancing the consequences of vaccination to overall health impacts, including understanding the impact of an altered mean age of infection for both chickenpox and shingles, would need to be considered prior to any vaccine introduction.

## Methods

### Data

Monthly clinical case reports for chickenpox and shingles were obtained from the Thailand Ministry of Health [52]. Population level data were acquired from the Thailand National Statistical Office [53]. Population and birth rate projection data were downloaded from the United Nations [54].

### Model and simulations

**Fig. 5:**
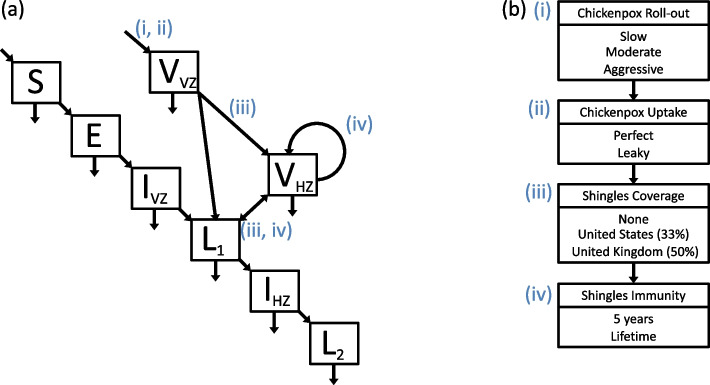
**a** Model schematic, which includes susceptible (*S*), exposed (*E*), vaccinated against chickenpox ($V_{VZ}$), infected with chickenpox ($I_{VZ}$), vaccinated against shingles ($V_{HZ}$), recovered from chickenpox ($L_1$), infected with shingles ($I_{HZ}$), and recovered from shingles ($L_2$) classes. Births entered the model through either the *S* class or the vaccinated against chickenpox ($V_{VZ}$) class depending on chickenpox vaccination (*i*, *ii*). Natural death occurred in all classes and is indicated by an arrow leaving each class at the bottom. **b** Vaccination simulations varied by chickenpox vaccination roll-out (*i*), chickenpox vaccination uptake (*ii*), shingles coverage (*iii*), and length of shingles immunity (*iv*). A model without any vaccination was also simulated where all values $(i)-(iv)$ were set to 0

Chickenpox and shingles data from Thailand were previously fit using a mechanistic model [38]. The model implemented here was similar, with two vaccinated classes added, one each for chickenpox and shingles (Fig. 5a). Full model equations can be seen in the supplemental information. In addition to a vaccine-free simulation, vaccination scenarios that considered different combinations of chickenpox vaccine roll-out, uptake of the chickenpox vaccine, shingles vaccine coverage, and length of immunity from the shingles vaccine were examined (Fig. 5b and Table S2). The only other change to the model [38] involved increasing the average lifespan to 76.6 years to account for improved healthcare, nutrition, and living conditions over the next hundred years.

To model the introduction of a chickenpox vaccine in Thailand, three vaccine roll-out scenarios were used: (i) slow — chickenpox vaccine uptake matching measles vaccine uptake in Thailand, which was introduced in 1984, (ii) moderate — chickenpox vaccine coverage matching hepatitis B vaccine coverage in Thailand, which began in 1992, and (iii) aggressive — a chickenpox vaccine uptake matching Japanese Encephalitis vaccine coverage in Thailand, which was introduced in 2006 (Fig. 1a) [39]. Chickenpox vaccine efficacy was allowed to vary, as the VZV vaccine has been shown to be leaky (i.e., it does not provide protective immunity to all vaccinated individuals). As an upper bound, the vaccine was considered perfect (i.e., immunization coverage equals vaccine coverage), while as a lower bound, only $79.5\%$ of vaccinated individuals became immune, which was the lowest efficacy found reported across multiple studies (range $79.5-92.6\%$) [55–57]. The low efficacy model was considered to be a combination of both primary vaccine failure and to roughly account for the waning of vaccine-derived immunity [58]. To keep the simulations conservative, only individuals entering the population as susceptibles via births were immunized against chickenpox (Fig. 5). This approach assumed no national immunization days or catch-up campaigns for children, teenagers, or adults. Chickenpox vaccine immunity was set to 20 years, the estimated mean length of immunity (10–20 years) plus the age at second dose (4–6 years) [59]. We also explored the dynamical impact of a longer lasting immunity form chickenpox vaccination (40 years) to examine the potential for a “better” vaccine. This immunity protected individuals from both chickenpox transmission and shingles reactivation. Upon loss of immunity, individuals became susceptible to shingles reactivation.

Shingles coverage was modeled as either non-existent (no shingles vaccination) or similar to the estimated coverage values from the USA ($33\%)$ [60] or the UK ($50\%$) [44–46]. Initial conditions for the vaccinated with shingles class ($V_{HZ}$) were considered to be either 0, $33\%$, or $50\%$ of the the recovered from chickenpox class ($L_1$), depending on shingles coverage. The model simulated shingles coverage by moving 0, $33\%$, or $50\%$ from the exiting vaccinated with chickenpox ($V_{VZ}$) and infected with chickenpox ($I_{VZ}$) classes to the $V_HZ$ class. Shingles length of immunity was considered to be either 5 years [61] or lifelong. Lifelong immunity could be interpreted as every individual who received the shingles vaccine will continue to receive their booster dose, removing the possibility of a $V_HZ$ individual moving into the latent and susceptible to shingles ($L_1$) class, or it could represent a new vaccine brought to market (Fig. 5). All scenarios that included shingles vaccination also included one form of chickenpox vaccination, though sensitivity analyses were performed where no chickenpox vaccination occurred. All models were run for 100 years and implemented with the R package *pomp* [62].

Though most of the results and discussion are focused on the vaccination scenarios listed in Fig. 5b, overall these simulations varied chickenpox vaccine coverage (0–100%), chickenpox vaccine effectiveness (79.5% or 100%), chickenpox vaccination duration of immunity (20 or 40 years), shingles vaccine coverage (0–100%), and shingles vaccination duration of immunity (5 years or lifetime).

## Supplementary Information

**Additional file 1:** Supplementary Materials.

## Data Availability

All data and code are available at https://www.kevinmbakker.com/data.html.

## Abbreviation

VZV: Varicella zoster virus
