# Supplementary material for: Identifying optimal vaccination scenarios to reduce varicella zoster virus transmission and reactivation
Source: BMC Med. 2022 Oct 8;20:387. doi: 10.1186/s12916-022-02534-7 (PMC9548166; doi:10.1186/s12916-022-02534-7)
Supplement: Supplementary file 1 — Additional file 1: Supplementary Materials. [file 12916_2022_2534_MOESM1_ESM.pdf]

Contents

1 Methods 2

1.1 Model 2

2 Results 7

Supporting Information: Identifying optimal vaccination scenarios to reduce varicella zoster virus transmission and reactivation

Kevin M Bakker[a], Marisa C Eisenberg[a,b], Robert J Woods[c] and Micaela E Martinez[d,e]

[a]Department of Epidemiology, University of Michigan, Ann Arbor, MI 48109, USA

[b]Department of Mathematics, University of Michigan, Ann Arbor, MI 48109, USA

[c]Division of Infectious Diseases, Department of Internal Medicine, University of Michigan, Ann Arbor, MI, 48109, USA

[d]Population Biology, Ecology and Evolutionary, Emory University, Atlanta, GA, 30322, USA

[e]University of Surrey, Faculty of Health and Medical Sciences, Guildford, United Kingdom

August 5, 2022

1 Methods

1.1 Model

To simulate VZV dynamics under various vaccination regimes, we expanded our susceptible, exposed, infected with chickenpox, latent (recovered from chickenpox), infected with shingles, and second latent (recovered from shingles) state model [1] to include chickenpox and shingles vaccinated classes (Fig 1). Population data for Thailand were collected from the United Nations, and interpolated for monthly births [2]. Model state transitions are shown in Eqs. 1—8. For each class A, pA represents the fraction who remain in the A class for the next time step (defined below). χ represents the effectiveness of a vaccine.

Parameter	Description	Units	Value	Range	Sources
VZ Spline 1	Chickenpox spline component	$\frac{1}{weeks}$	4.15	(3.88, 5.15)	Fitted
VZ Spline 2	Chickenpox spline component	$\frac{1}{weeks}$	3.40	(3.32,4.64)	Fitted
VZ Spline 3	Chickenpox spline component	$\frac{1}{weeks}$	0.94	(0.90,1.27)	Fitted
VZ Spline 4	Chickenpox spline component	$\frac{1}{weeks}$	1.85	(1.75,2.43)	Fitted
VZ Spline 5	Chickenpox spline component	$\frac{1}{weeks}$	2.30	(2.20,2.89)	Fitted
VZ Spline 6	Chickenpox spline component	$\frac{1}{weeks}$	1.56	(1.46,2.08)	Fitted
HZ Spline 1	Shingles spline component	$\frac{1}{weeks}$	1.93	(1.31,1.98)	Fitted
HZ Spline 2	Shingles spline component	$\frac{1}{weeks}$	2.47	(1.76,2.55)	Fitted
HZ Spline 3	Shingles spline component	$\frac{1}{weeks}$	2.46	(1.67,2.47)	Fitted
HZ Spline 4	Shingles spline component	$\frac{1}{weeks}$	2.83	(1.95,2.90)	Fitted
HZ Spline 5	Shingles spline component	$\frac{1}{weeks}$	2.41	(1.69,2.50)	Fitted
HZ Spline 6	Shingles spline component	$\frac{1}{weeks}$	2.22	(1.51,2.22)	Fitted
ρ^{VZ}	Chickenpox reporting rate	Percent	99.5	(77.56,100)	Fitted
M1	Time-varying report rate parameter for shingles	NA	3.25e-5	(3.10e-5,3.38e-5)	Fitted
M2	Baseline report rate for shingles	NA	0.003	(1.6e-14,154)	Fitted
β_{SD}	Standard deviation for process noise	NA	0.24	(0.18,0.27)	Fitted
ψ	Exogenous boosting	NA	1	NA	Fitted
$\frac{1}{\delta}$	Lifespan	Weeks	$73 * 52$	NA	[2]
$\frac{1}{\iota}$	Shingles Reactivation Probability	Weeks	$60 * 52$	NA	[3, 4]
$\frac{1}{\xi}$	Length of chickenpox immunity	Weeks	20 (or 40)*52	NA	[5]
$\frac{1}{\sigma}$	Length of shingles immunity	Weeks	5 years or lifetime	NA	[6]
τ	Chickenpox vaccination level	Value	$0 - 100$	NA	[6]
χ	Shingles vaccination level	value	$0 - 1$	NA	[6]

Table S1: Parameter Table of fitted MLEs from [1], with additional parameters for vaccination simulations.

$$S_{t+1} = (1 - \tau) * \mu_t + (S_t \cdot pS) \tag{1}$$

where μ_t is the number of children born at time, t (treated as a known forcing function based on data). τ represented the chickenpox vaccination rate. Below are the transitions for states E-L2;

$$E_{t+1} = S_t * (1 - pS) \left(\frac{\lambda}{\lambda + \delta} \right) + (E_t \cdot pE) \tag{2}$$

$$I_{VZ_{t+1}} = E_t * (1 - pE) \left(\frac{\phi}{\phi + \delta} \right) + (I_{VZ_t} \cdot pI_{VZ}) \tag{3}$$

$$\begin{aligned} L_{1_{t+1}} = (1 - \chi) * I_{VZ_t} * (1 - pI_{VZ}) \left(\frac{\gamma}{\gamma + \delta} \right) + (1 - \chi) * V_{VZ_t} * (1 - pV_{VZ}) \left(\frac{\xi}{\xi + \delta} \right) + \\ (1 - \chi) * V_{HZ_t} * (1 - pV_{HZ}) \left(\frac{\sigma}{\sigma + \delta} \right) + (L_{1_t} \cdot pL_1) \end{aligned} \tag{4}$$

$$I_{HZ_{t+1}} = L_{1_t} * (1 - pL_1) \left(\frac{\iota\kappa\psi}{\iota\kappa\psi + \delta} \right) + (I_{HZ_t} \cdot pI_{HZ}) \tag{5}$$

$$L_{2_{t+1}} = I_{HZ_t} * (1 - pI_{HZ}) \left(\frac{\gamma}{\gamma + \delta} \right) + (L_{2_t} \cdot pL_2). \tag{6}$$

$$V_{VZ_{t+1}} = \tau * \mu_t + (V_{VZ_t} \cdot pV_{VZ}). \tag{7}$$

$$\begin{aligned} V_{HZ_{t+1}} = \chi * I_{VZ_t} * (1 - pI_{VZ}) \left(\frac{\gamma}{\gamma + \delta} \right) + \chi * V_{VZ_t} * (1 - pV_{VZ}) \left(\frac{\xi}{\xi + \delta} \right) + \\ \chi * V_{HZ_t} * (1 - pV_{HZ}) \left(\frac{\sigma}{\sigma + \delta} \right) + (L_{1_t} \cdot pL_1) \end{aligned} \tag{8}$$

New infections for chickenpox at each time step were recorded as

$$I_{VZ_{new}} = E_t * (1 - pE) \left(\frac{\phi}{\phi + \delta} \right) \tag{9}$$

while new shingles infections were recorded as

$$I_{HZ_{new}} = L_{1_t} * (1 - pL_1) \left(\frac{\iota\kappa\psi}{\iota\kappa\psi + \delta} \right). \tag{10}$$

The above difference equations for each state are displayed in Fig 1, using the transition rates from Eqs. 11, 14—16, & 18—21. The generalized model transition probabilities followed a Poisson process,

where the fraction of those who remained in the susceptible state (pS) was modeled as

$$pS = e^{-(\lambda+\delta)} \tag{11}$$

where λ is the force of infection and δ is the death rate, which was assumed to be constant across all model states and set for an average lifespan of 73 years [2]. The force of infection, λ was estimated as

$$\lambda = \beta \left(\frac{(I_{VZ}) + (\omega I_{HZ})}{N} \right)^{\alpha} \epsilon \tag{12}$$

where β was the time-varying seasonal force of infection for chickenpox. I_{VZ} was the number of current individuals infected with chickenpox, ω was a scalar for the relative infectiousness of shingles - allowing for reactivated shingles individuals to infect a susceptible individual, I_{HZ} was the number of individuals with reactivated shingles, N was the total population, α was a scalar for the force of infection [7], and ϵ was a noise term which acted as environmental stochasticity. ϵ, was drawn from a gamma distribution with a mean of 1 and variance θ (process noise dispersion parameter) [8].

A B-spline was used to fit β. Biologically, this represents the hypothesis that chickenpox is driven by a combination of seasonal factors (including school terms wherein students have increased contact rates and other seasonal factors).

$$\beta = \exp \sum_{i=1}^{6} q_i \zeta_{A_{i_t}} \tag{13}$$

where β is the seasonal forcing for chickenpox and each ζ_A is a periodic B-spline basis with 1 year period. β is summarized in table S1 as VZ splines 1-6. The fraction of those who remained in the exposed state were modeled as

$$pE = e^{-(\phi+\delta)} \tag{14}$$

where ϕ was a rate at which an individual became infectious after being exposed to chickenpox (parameter scaled to 1/2 week). The fraction of those who remained in the infected with chickenpox state I_{VZ} were modeled as

$$pIVZ = e^{-(\gamma+\delta)} \tag{15}$$

where γ was a fixed rate in which individuals recovered from chickenpox (parameter scaled to 7 days). The fraction of those who remained in the first latent state, but remained susceptible to shingles reactivation were modeled as

$$pL1 = e^{-(\iota\kappa\psi+\delta)} \tag{16}$$

where ι was a fixed parameter which represented the fraction of those infected with chickenpox that would reactivate later in life as shingles (parameter scaled for a mean age 60 of years), κ is the seasonal reactivation rate of shingles, and ψ is the time-varying immunity boosting from chickenpox infections (see below for both κ and ψ model combinations, also Table S1 and Fig 2). κ was modeled as a B-spline, similar to chickenpox:

$$\kappa = \exp \sum_{i=1}^{6} q_i \zeta_{B_{i_t}} \tag{17}$$

where each ζ_B is a different (from ζ_A) B-spline basis with 1 year period. κ is summarized in table S1 as HZ splines 1-6. All parameters were estimated using maximum likelihood by iterated particle filtering (MIF) in the R-package [9, 10].

The fraction of those who remained infected with shingles were modeled as

$$pIHZ = e^{-(\gamma+\delta)} \tag{18}$$

with the same recovery rate, γ (parameter scaled to 7 days), as chickenpox, while the fraction of those who remained in the second latent state were modeled as

$$pL2 = e^{-(\delta)} \tag{19}$$

where natural death was the only exit from the second latent state.

The fraction of those who remained in the chickenpox vaccinated class was modeled as

$$pV_{VZ} = e^{-(\xi+\delta)} \tag{20}$$

where ξ represented the length of immunity to chickenpox vaccination, which was set to 20 years [5].

The fraction of those who remained in the shingles vaccinated class was modeled as

$$pV_{HZ} = e^{-(\sigma+\delta)} \tag{21}$$

where σ represented the length of immunity to shingles vaccination, which was set to either 5 years [6] or lifetime.

2 Results

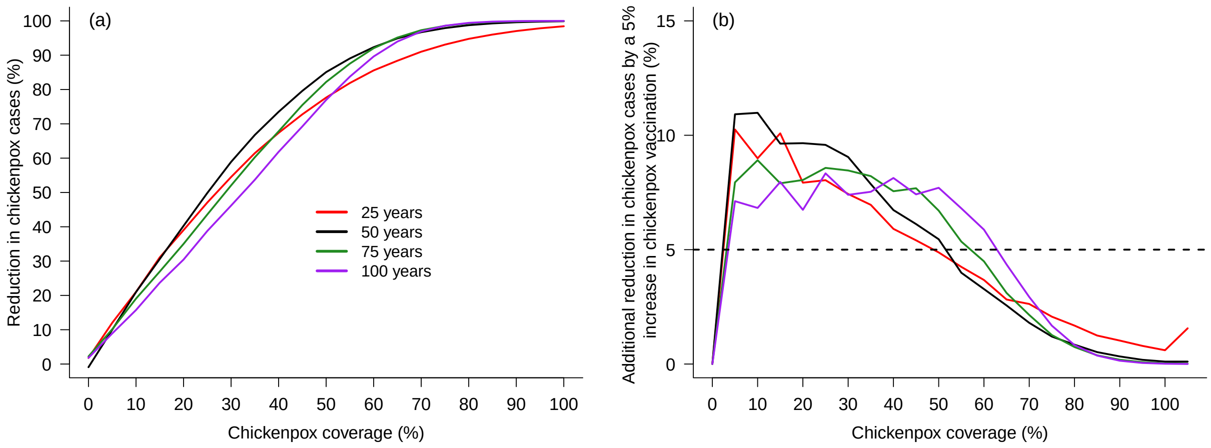

Figure S1: Impact of chickenpox vaccination. (a) Chickenpox coverage is shown on the x-axis and the total reduction in chickenpox cases on the y-axis. The lines represent case reductions at 25 years (red), 50 years (black), 75 years (green), and 100 years (purple). (b) Percentage of additional chickenpox cases prevented by increasing coverage by 5% (that is, the rate of change in chickenpox reduction from (a). Dotted line at 5% identifies where you would achieve a greater than 5% reduction in cases for 5% additional coverage (above the line) and where you would achieve less than a 5% reduction in cases (below the dotted line).

Table S2: Summary of the total cases over 100 years of all models against the null model lacking vaccination for chickenpox or shingles. ID is the model number correlating to Fig 1. Model ID is a quick reference to the model combinations explained in the next four columns. VZ Roll-out is the chickenpox vaccination coverage (slow - measles, moderate - hepatitis B, aggressive - Japanese Encephalitis), VZ uptake is the uptake level for the chickenpox vaccine, HZ coverage is the shingles vaccination coverage, and HZ immunity is the length of immunity provided by shingles vaccination. All models were compared to the number of cases with no chickenpox or shingles vaccination (top row). % of VZ cases is the percentage of chickenpox cases in that model scenario compared to the scenario without any vaccination over the entire 100 years (top row). % of HZ cases is the percentage of chickenpox cases in that model scenario compared to the scenario without any vaccination over the entire 100 years (top row).

ID	VZ Roll-out	VZ Uptake	HZ Coverage	HZ Immunity	% of VZ Cases	% of HZ Cases
NA_NA_NA_NA	NA	NA	NA	NA	100%	100%
S_P_NA_NA	Slow	Perfect	NA	NA	12.9%	142.7%
S_P_US_5	Slow	Perfect	33%	5	12.8%	135.2%
S_P_US_L	Slow	Perfect	33%	Life	12.9%	95.6%
S_P_UK_5	Slow	Perfect	50%	5	12.9%	128.2%
S_P_UK_L	Slow	Perfect	50%	Life	12.8%	71.3%
S_L_NA_NA	Slow	Leaky	NA	NA	16.4%	121.1%
S_L_US_5	Slow	Leaky	33%	5	16.5%	114.9%
S_L_US_L	Slow	Leaky	33%	Life	16.3%	81.1%
S_L_UK_5	Slow	Leaky	50%	5	16.3%	108.9%
S_L_UK_L	Slow	Leaky	50%	Life	16.4%	60.5%
M_P_NA_NA	Moderate	Perfect	NA	NA	8.9%	149.4%
M_P_US_5	Moderate	Perfect	33%	5	8.9%	141.7%
M_P_US_L	Moderate	Perfect	33%	Life	9.0%	100.1%
M_P_UK_5	Moderate	Perfect	50%	5	9.1%	134.6%
M_P_UK_L	Moderate	Perfect	50%	Life	9.1%	74.8%
M_L_NA_NA	Moderate	Leaky	NA	NA	12.1%	125.1%
M_L_US_5	Moderate	Leaky	33%	5	12.1%	118.8%
M_L_US_L	Moderate	Leaky	33%	Life	12.2%	83.9%
M_L_UK_5	Moderate	Leaky	50%	5	12.2%	112.9%
M_L_UK_L	Moderate	Leaky	50%	Life	12.2%	62.6%
A_P_NA_NA	Aggressive	Perfect	NA	NA	7.1%	147.3%
A_P_US_5	Aggressive	Perfect	33%	5	7.0%	139.7%
A_P_US_L	Aggressive	Perfect	33%	Life	7.2%	98.7%
A_P_UK_5	Aggressive	Perfect	50%	5	7.0%	132.6%
A_P_UK_L	Aggressive	Perfect	50%	Life	7.1%	73.6%
A_L_NA_NA	Aggressive	Leaky	NA	NA	10.5%	123.2%
A_L_US_5	Aggressive	Leaky	33%	5	10.6%	117.1%
A_L_US_L	Aggressive	Leaky	33%	Life	10.5%	82.5%
A_L_UK_5	Aggressive	Leaky	50%	5	10.6%	111.3%
A_L_UK_L	Aggressive	Leaky	50%	Life	10.5%	61.6%

Table S3: Summary of the total cases in 25 year intervals for all models against the null model lacking vaccination for chickenpox or shingles. ID is the model number correlating to Fig 1 & Table S2. All models were compared to the number of cases with no chickenpox or shingles vaccination (top row). VZ is the percentage of chickenpox cases in that model scenario compared to the scenario without any vaccination (top row). HZ is the percentage of shingles cases in that model scenario compared to the scenario without any vaccination (top row). Greyed rows represent rows that never experienced an increase in shingles cases compared to the no vaccination model.

% of cases								
ID	Years 1-25		Years 26-50		Years 51-75		Years 76-100	
	VZ	HZ	VZ	HZ	VZ	HZ	VZ	HZ
NA_NA_NA_NA	100%	100%	100%	100%	100%	100%	100%	100%
S_P_NA_NA	42.8%	101%	1.8%	150.6%	0.2%	164.1%	0.4%	145.7%
S_P_US_5	42.4%	87.0%	1.8%	141.1%	0.2%	158.5%	0.4%	142.6%
S_P_US_L	42.7%	67.4%	1.8%	100.9%	0.2%	110.0%	0.4%	97.6%
S_P_UK_5	42.7%	76.7%	1.8%	132.0%	0.2%	152.5%	0.4%	139.1%
S_P_UK_L	42.6%	50.2%	1.8%	75.3%	0.2%	82.0%	0.4%	72.9%
S_L_NA_NA	49.5%	97.9%	5.7%	129.5%	1.9%	134.3%	1.3%	117.8%
S_L_US_5	49.6%	85.3%	5.7%	122.4%	1.9%	130.1%	1.3%	115.3%
S_L_US_L	49.2%	65.2%	5.8%	86.7%	1.8%	90.0%	1.3%	78.9%
S_L_UK_5	49.0%	74.4%	5.8%	114.9%	1.8%	125.6%	1.3%	112.7%
S_L_UK_L	49.3%	48.8%	5.7%	64.7%	1.9%	67.1%	1.3%	58.9%
M_P_NA_NA	29.9%	105.8%	0.8%	164.0%	0.1%	170.4%	0.2%	147.7%
M_P_US_5	30.0%	91.6%	0.8%	154.0%	0.1%	165.2%	0.2%	145.0%
M_P_US_L	30.2%	71.1%	0.8%	110.0%	0.1%	114.2%	0.2%	99.0%
M_P_UK_5	30.6%	80.1%	0.8%	144.1%	0.1%	159.5%	0.2%	141.6%
M_P_UK_L	30.6%	53.3%	0.8%	82.1%	0.1%	85.2%	0.2%	73.9%
M_L_NA_NA	37.1%	100.2%	3.6%	138.1%	1.3%	138.3%	1.0%	118.9%
M_L_US_5	36.9%	87.0%	3.6%	130.4%	1.3%	134.4%	1.0%	116.6%
M_L_US_L	37.3%	67.3%	3.6%	92.6%	1.3%	92.7%	1.0%	79.6%
M_L_UK_5	37.3%	76.6%	3.6%	122.7%	1.3%	130.0%	1.0%	114.1%
M_L_UK_L	37.4%	50.3%	3.6%	69.1%	1.3%	69.2%	1.0%	59.4%
A_P_NA_NA	23.7%	107.8%	0.8%	164.1%	0.1%	166.3%	0.3%	142.6%
A_P_US_5	23.5%	92.3%	0.8%	154.3%	0.1%	161.4%	0.3%	139.9%
A_P_US_L	24.0%	72.4%	0.8%	110.0%	0.1%	111.4%	0.3%	95.6%
A_P_UK_5	23.5%	81.1%	0.8%	144.5%	0.1%	156.1%	0.3%	136.9%
A_P_UK_L	23.6%	53.4%	0.8%	82.0%	0.1%	83.1%	0.3%	71.3%
A_L_NA_NA	31.0%	100.7%	3.7%	137.7%	1.6%	135.1%	1.4%	115.1%
A_L_US_5	31.0%	87.6%	3.7%	130.3%	1.6%	131.5%	1.4%	112.9%
A_L_US_L	30.9%	67.4%	3.7%	92.2%	1.6%	90.5%	1.4%	77.1%
A_L_UK_5	31.1%	76.7%	3.7%	122.7%	1.6%	127.4%	1.4%	110.6%
A_L_UK_L	31.0%	50.4%	3.7%	68.9%	1.6%	67.6%	1.4%	57.5%

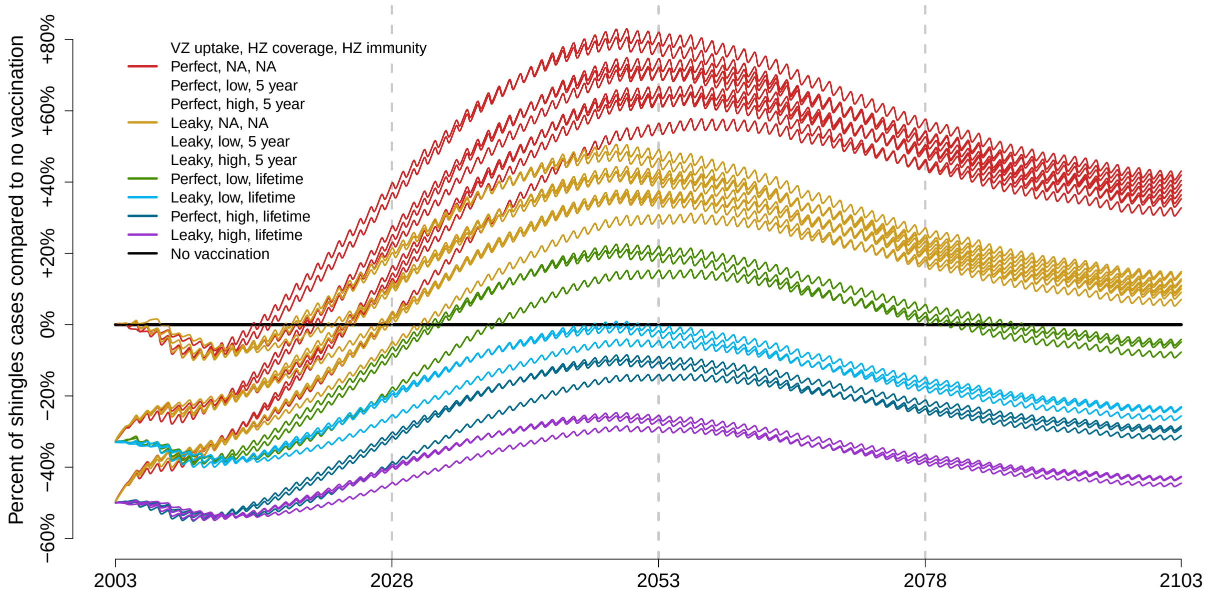

Figure S2: Percent of shingles cases by month of each model simulation compared to the null model with no vaccination (black). Models that offered no shingles vaccination or only 5-years of shingles immunity from vaccination, whether with perfect (red) or leaky (yellow) chickenpox uptake resulted in an increase in shingles cases compared to no vaccination at all. Lifetime shingles immunity from vaccination with low shingles coverage and perfect (green) or leaky (light blue) chickenpox uptake, resulted in reduced shingles cases. Lifetime shingles immunity paired with high shingles coverage rates with perfect (dark blue) or leaky (purple) chickenpox uptake resulted in the largest reduction in shingles cases.

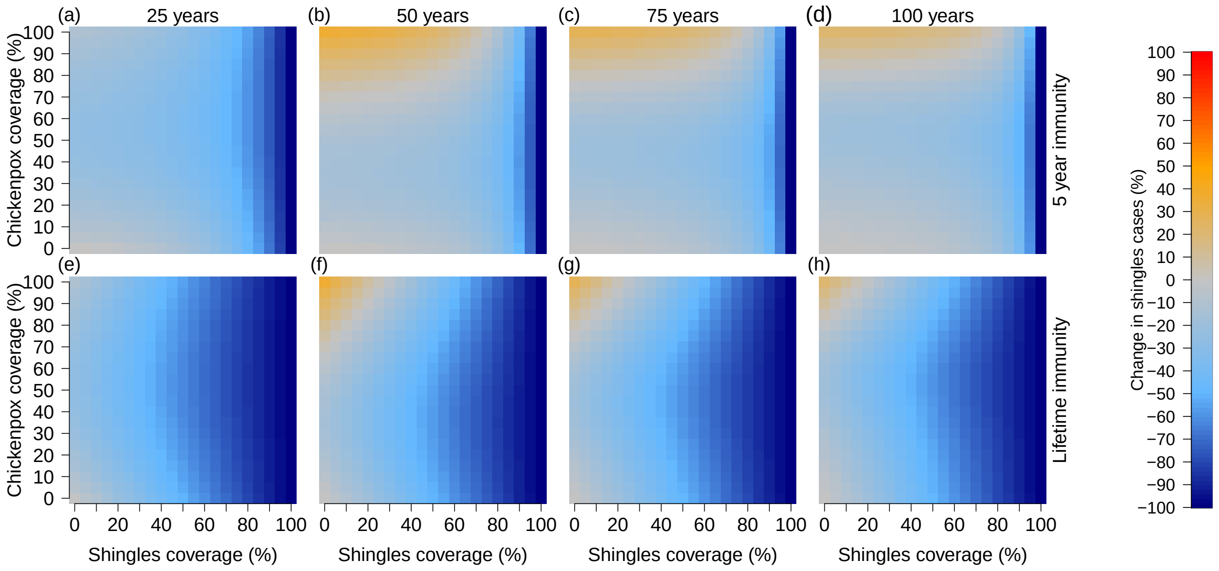

Figure S3: Percent change in shingles cases compared to no vaccination, using a 40 year protection from chickenpox vaccination. Top row (a—d) simulations provided 5 years of immunity from shingles vaccination and bottom row (e—h) simulations provided lifetime immunity from shingles vaccination. Each column represents the number of years after vaccine introduction; (a & e) 25 years, (b & f) 50 years, (c & g) 75 years, and (d & h) 100 years. Shingles and chickenpox vaccine coverage (%) are shown on the x- and y-axes. Color scale on the right indicates the largest decrease in shingles cases can be seen in dark blue, while the largest increase in shingles cases can be seen in red.

References

1. Bakker, K. M, Eisenberg, M. C, Woods, R, & Martinez, M. E. (2021) Exploring the seasonal drivers of varicella zoster virus transmission and reactivation. *American Journal of Epidemiology* **190**, 1814–1820.

2. UN. (2015) http://esa.un.org/unpd/wpp/ Accessed June 18, 2015.

3. Thomas, S & Hall, A. (2001) What does epidemiology tell us about risk factors for herpes zoster? *The Lancet* **4**, 26–33.

4. Donahue, J, Choo, P, Manson, J, & Platt, R. (1995) The incidence of herpes zoster. *Archives of Internal Medicine* **155**, 1605–1609.

5. CDC. (2020) About the Varicella Vaccine. Accessed May 11, 2020.

6. CDC. (2020) Zostovax. Accessed May 11, 2020.

7. Stroud, P. D, Sydoriak, S. J, Riese, J. M, Smith, J. P, Mniszewski, S. M, & Romero, P. R. (2006) Semi-empirical power-law scaling of new infection rate to model epidemic dynamics with inhomogeneous mixing. *Mathematical biosciences* **203**, 301–318.

8. Martinez-Bakker, M, Rohani, P, & King, A. A. (2015) Unraveling the transmission dynamics of polio. *PLoS Biology* **13**, e1002172.

9. King, A. A, Nguyen, D, & Ionides, E. L. (2016) Statistical inference for partially observed Markov processes via the R package pomp. *Journal of Statistical Software* **69**, 1–43.

10. King, A. A, Ionides, E. L, Bretó, C. M, Ellner, S. P, Ferrari, M. J, Kendall, B. E, Lavine, M, Nguyen, D, Reuman, D. C, Wearing, H, & Wood, S. N. (2020) *pomp: Statistical Inference for Partially Observed Markov Processes*. R package, version 2.5.
